# Neuroendocrine Stress Response in Acute Stroke: Physiological and Clinical Perspectives

**DOI:** 10.7759/cureus.103236

**Published:** 2026-02-08

**Authors:** Isha Atam, Shraddha Singh, Satish Kumar, Abhishek K Singh, Danish Rastogi

**Affiliations:** 1 Physiology, King George's Medical University, Lucknow, IND; 2 Medicine, King George's Medical University, Lucknow, IND

**Keywords:** acute ischemic stroke, ambulatory blood pressure monitoring, autonomic dysfunction, circadian rhythm, cortisol, hypothalamic pituitary adrenal axis, melatonin

## Abstract

Acute ischemic stroke constitutes a significant systemic stress event that initiates a complex cascade of neuroendocrine and autonomic responses. Central to this process is activation of the hypothalamic-pituitary-adrenal (HPA) axis and the sympathetic nervous system, reflecting the brain’s integrated response to acute cerebral injury. These changes are accompanied by widespread alterations in physiological regulation, including endocrine signaling, autonomic balance, circadian organization, and cardiovascular control. Understanding the nature and coordination of these responses is important for gaining insight into the broader pathophysiological impact of acute ischemic stroke beyond focal neuronal damage. This narrative review provides an overview of the mechanisms underlying HPA axis activation, circadian rhythm dysregulation, and autonomic dysfunction in the setting of acute ischemic stroke. Particular emphasis is placed on the interactions between neuroendocrine signaling and cardiovascular regulation, highlighting how disruptions in these systems may reflect the severity of systemic stress and autonomic imbalance. The review also discusses the relevance of neuroendocrine and autonomic biomarkers, including cortisol, melatonin, and ambulatory blood pressure parameters, as integrative indicators of physiological stress, autonomic regulation, and recovery patterns in acute stroke.

## Introduction and background

Acute ischemic stroke represents a profound physiological stressor that activates the hypothalamic-pituitary-adrenal (HPA) axis and the sympathetic nervous system, leading to marked neuroendocrine and autonomic alterations [1,2]. These changes include elevated cortisol levels, suppression of melatonin secretion, disruption of circadian rhythms, and abnormalities in blood pressure regulation [3-5]. While these responses are initially adaptive, excessive or sustained neuroendocrine activation can contribute to secondary brain injury, immune dysregulation, autonomic imbalance, and adverse clinical outcomes [6-8]. Understanding the neuroendocrine stress response in acute stroke is therefore important from both physiological and clinical perspectives. This narrative review summarizes current evidence on HPA axis activation, circadian rhythm disruption, autonomic dysfunction, and their interactions with cardiovascular regulation, highlighting the clinical relevance of neuroendocrine biomarkers such as cortisol, melatonin, and ambulatory blood pressure parameters. It focuses specifically on acute ischemic stroke, excluding hemorrhagic stroke and transient ischemic attack. The term “acute” refers to the early phase following symptom onset, generally encompassing the first several days (particularly the first 24-72 hours), during which neuroendocrine and autonomic alterations are most dynamic.

Stroke is a leading cause of mortality and long-term disability worldwide, with acute ischemic stroke accounting for approximately 80% of all cases [9]. Beyond focal cerebral injury, stroke induces widespread systemic disturbances affecting the cardiovascular, endocrine, immune, and autonomic systems [10,11]. Among these, the neuroendocrine stress response plays a central role in shaping acute pathophysiology and influencing clinical outcomes. [2,6]

The brain is the primary regulator of endocrine and autonomic function. Acute cerebral ischemia disrupts neural networks involving the hypothalamus, limbic system, insular cortex, and brainstem, triggering activation of the HPA axis and sympathetic nervous system [12,13]. This leads to increased secretion of stress hormones such as cortisol and catecholamines, suppression of melatonin, loss of circadian rhythmicity, and abnormal blood pressure patterns [4,5,14]. Although these responses aim to preserve homeostasis, their dysregulation may exacerbate neuronal injury and worsen prognosis [6-8].

## Review

This narrative review was conducted to synthesize current evidence on neuroendocrine and autonomic alterations in acute ischemic stroke. A structured literature search was performed using PubMed/MEDLINE and Google Scholar for articles published in English up to December 2025. Search terms included combinations of “acute ischemic stroke”, “cortisol”, “melatonin”, “hypothalamic-pituitary-adrenal axis”, “autonomic dysfunction”, and “ambulatory blood pressure monitoring”.

Studies were selected based on relevance to neuroendocrine mechanisms and clinical outcomes in the acute phase of ischemic stroke. Both clinical and experimental studies were considered. As this is a narrative review, formal systematic review methodology was not applied.

Neuroendocrine stress response: physiological overview

Stress and Homeostasis

Stress is broadly defined as any real or perceived challenge that threatens the stability of internal physiological conditions, commonly referred to as homeostasis. In response to such challenges, the body initiates a set of coordinated adaptive mechanisms designed to restore equilibrium and ensure survival. These responses are mediated predominantly by the neuroendocrine and autonomic systems, which function as the primary interface between the brain and peripheral organs during stressful conditions. Activation of these systems enables rapid communication and integration of signals across multiple organ systems, allowing the organism to mount an effective response to environmental, physiological, or psychological stressors.

The neuroendocrine response to stress involves activation of hormonal pathways that regulate energy metabolism, immune activity, cardiovascular function, and circadian organization. Simultaneously, the autonomic nervous system modulates visceral functions through dynamic adjustments in sympathetic and parasympathetic activity. Together, these systems facilitate short-term adaptive changes that support homeostatic restoration. However, when stress is severe, prolonged, or dysregulated, the same mechanisms may contribute to maladaptive physiological consequences and disease progression [1,2]. The HPA axis and the autonomic nervous system serve as the principal effectors of the stress response, functioning as integrated regulatory systems that translate central neural signals into coordinated peripheral actions. These systems continuously process information from cortical, limbic, and brainstem regions, as well as from peripheral sensory inputs, allowing the organism to detect and respond to internal and external stressors. Through this integration, they orchestrate adaptive changes in hormonal secretion, cardiovascular regulation, metabolic activity, immune function, and circadian organization, all of which contribute to the preservation of internal stability.

By linking higher brain centers with endocrine glands and autonomic effector organs, the HPA axis and autonomic nervous system ensure rapid and sustained modulation of physiological processes during stress. While these responses are essential for maintaining homeostasis under acute challenges, dysregulation of these systems can disrupt physiological balance and contribute to pathological states, particularly in conditions characterized by acute or chronic systemic stress [15].

Hypothalamic-Pituitary-Adrenal Axis

Activation of the HPA axis is initiated by the release of corticotropin-releasing hormone from the hypothalamus in response to stress-related signals. This hormone acts on the anterior pituitary gland, stimulating the synthesis and secretion of adrenocorticotropic hormone into the systemic circulation. Adrenocorticotropic hormone subsequently acts on the adrenal cortex, promoting the production and release of cortisol, the principal glucocorticoid involved in the stress response. Cortisol exerts widespread effects on multiple organ systems, influencing energy metabolism, vascular tone, immune regulation, and central nervous system function. Through these actions, activation of the HPA axis plays a central role in coordinating the body’s adaptive response to stress and maintaining physiological homeostasis [2,16]. Cortisol exerts wide-ranging metabolic, cardiovascular, and immunomodulatory effects that are essential for maintaining physiological homeostasis, particularly during periods of stress. It regulates glucose and lipid metabolism, influences vascular tone and blood pressure, and modulates immune and inflammatory responses. Under normal conditions, cortisol secretion follows a distinct circadian rhythm governed by the suprachiasmatic nucleus of the hypothalamus, the central pacemaker of the circadian system. Cortisol levels typically peak in the early morning hours, facilitating arousal and metabolic readiness, and gradually decline throughout the day to reach a nadir during the nighttime. This rhythmic pattern is critical for synchronizing endocrine function with sleep-wake cycles and other circadian-regulated physiological processes [17,18]. During acute stress, normal circadian regulation of cortisol secretion can be overridden by stress-responsive neural and hormonal signaling pathways. Intense or sudden stressors activate hypothalamic and brainstem circuits that drive corticotropin-releasing hormone release independently of circadian input from the suprachiasmatic nucleus. As a result, cortisol secretion becomes dominated by stress-related signaling rather than by time-of-day cues, leading to sustained or inappropriately elevated cortisol levels. This alteration reflects a shift from rhythmic endocrine regulation to a continuous stress-response mode aimed at ensuring metabolic and cardiovascular support during acute challenges. While this response may be adaptive in the short term, prolonged elevation of cortisol can disrupt circadian organization and contribute to adverse physiological effects [16,19].

HPA Axis Activation in Acute Ischemic Stroke

Acute ischemic stroke acts as a potent activator of the HPA axis due to the sudden disruption of cerebral homeostasis and the intense physiological stress it imposes. Ischemic injury to the brain triggers afferent signaling from cortical, limbic, and brainstem regions involved in stress perception and autonomic regulation, leading to robust activation of hypothalamic stress pathways. In addition, neuroinflammation, metabolic imbalance, and altered cerebral perfusion further amplify HPA axis activity. This heightened activation results in increased cortisol secretion, reflecting the systemic stress burden associated with acute cerebral ischemia and contributing to widespread neuroendocrine and autonomic alterations observed during the acute phase of stroke [6,7]. Elevated cortisol levels in serum, saliva, and urine have consistently been observed during the acute phase of ischemic stroke, reflecting robust activation of the HPA axis in response to cerebral injury. These increases are evident across multiple biological matrices, underscoring the systemic nature of the stress response following stroke. Higher cortisol concentrations have been shown to correlate positively with infarct volume, indicating a relationship between the extent of cerebral tissue injury and the magnitude of neuroendocrine activation. In addition, elevated cortisol levels are associated with greater neurological deficit severity, as assessed by clinical scoring systems, suggesting that cortisol may serve as a marker of disease burden and physiological stress. Importantly, persistently elevated cortisol levels have also been linked to increased mortality, highlighting their potential prognostic significance. Together, these observations support the role of cortisol as both a reflection of acute stress intensity and a potential indicator of clinical outcome in patients with acute ischemic stroke [6,20,21]. Patients with ischemic lesions involving key autonomic regulatory regions such as the insular cortex, hypothalamus, or brainstem often exhibit exaggerated cortisol responses during the acute phase of stroke. These brain regions are integral components of the central autonomic network and play a crucial role in integrating sensory, emotional, and visceral information with neuroendocrine output. Damage to these structures disrupts normal inhibitory and excitatory control over hypothalamic stress pathways, leading to amplified activation of the HPA axis. The resulting heightened cortisol secretion underscores the importance of central autonomic networks in modulating neuroendocrine regulation and illustrates how lesion location can influence the magnitude of systemic stress responses following acute cerebral ischemia [12,22].

Excess cortisol during the acute phase of ischemic stroke may exacerbate neuronal injury through multiple interconnected mechanisms. Elevated cortisol levels can enhance excitotoxicity by increasing glutamate release and impairing neuronal calcium homeostasis, thereby amplifying ischemia-induced neuronal damage. Cortisol also promotes oxidative stress by altering mitochondrial function and increasing the generation of reactive oxygen species, which further contributes to cellular injury. In addition, cortisol-induced endothelial dysfunction can impair cerebrovascular regulation, reduce nitric oxide bioavailability, and compromise cerebral microcirculation. Disruption of endothelial integrity may also weaken the blood-brain barrier, facilitating the extravasation of inflammatory mediators and neurotoxic substances into the brain parenchyma. Collectively, these effects suggest that excessive cortisol exposure can intensify secondary brain injury processes and negatively influence neurological outcomes following acute ischemic stroke [23,24]. Furthermore, sustained hypercortisolemia following acute ischemic stroke can lead to significant immunosuppression, impairing both innate and adaptive immune responses. Elevated cortisol levels inhibit the production of pro-inflammatory cytokines, reduce lymphocyte proliferation, and suppress natural killer cell activity, thereby weakening host defense mechanisms. This cortisol-mediated immune dysregulation increases susceptibility to post-stroke infections, particularly pneumonia and urinary tract infections, which are among the most common complications in the acute and subacute phases of stroke. Such infections contribute substantially to prolonged hospitalization, delayed neurological recovery, and increased morbidity and mortality. The association between hypercortisolemia, immune suppression, and infectious complications highlights the broader systemic consequences of neuroendocrine stress activation and underscores the importance of recognizing cortisol dysregulation as a clinically relevant factor in post-stroke outcomes [25,26].

Circadian rhythm disruption and melatonin suppression

Melatonin Physiology

Melatonin, a hormone secreted primarily by the pineal gland, plays a central role in the regulation of circadian rhythms and the synchronization of the sleep-wake cycle. Its secretion is tightly controlled by the circadian pacemaker located in the suprachiasmatic nucleus of the hypothalamus and is modulated by environmental light-dark cues transmitted through the retinohypothalamic pathway. Under normal physiological conditions, melatonin levels rise during the evening, peak at night, and decline in the early morning, providing a hormonal signal of darkness that promotes sleep initiation and maintenance. Through its actions on central and peripheral targets, melatonin coordinates circadian timing across multiple physiological systems, contributing to sleep regulation, metabolic homeostasis, and overall neuroendocrine balance [27]. In addition to its role as a chronobiotic hormone, melatonin exhibits a broad range of antioxidant, anti-inflammatory, and neuroprotective properties that contribute to cellular and vascular homeostasis. Melatonin acts as a potent free radical scavenger, directly neutralizing reactive oxygen and nitrogen species and enhancing the activity of endogenous antioxidant enzymes. Through these actions, it helps limit oxidative damage to neuronal and endothelial cells. Melatonin also modulates inflammatory pathways by reducing the production of pro-inflammatory cytokines and inhibiting microglial activation, thereby attenuating neuroinflammatory responses. Furthermore, melatonin exerts sympatholytic effects by suppressing sympathetic nervous system activity and enhancing parasympathetic tone, which contributes to improved autonomic balance. Its vasodilatory actions, mediated through endothelial mechanisms and nitric oxide signaling, support vascular relaxation and cerebral blood flow regulation. Collectively, these properties underscore the multifaceted role of melatonin in protecting neural and cardiovascular function, particularly under conditions of physiological stress [28,29]. Under physiological conditions, melatonin secretion exhibits a well-defined nocturnal rise, initiated shortly after the onset of darkness and maintained throughout the night, with minimal secretion during daylight hours. This rhythmic pattern is tightly regulated by the central circadian pacemaker in the suprachiasmatic nucleus and is synchronized to the light-dark cycle via retinal photic input. The nocturnal surge in melatonin serves as an internal signal of biological night, coordinating sleep propensity, autonomic tone, endocrine rhythms, and cardiovascular function. Preservation of this pattern reflects intact circadian organization, whereas attenuation, phase shifts, or loss of nocturnal melatonin secretion indicate circadian misalignment and neuroendocrine dysregulation [27].

Effect of Stroke on Melatonin Secretion

Acute stroke interferes with normal circadian regulation through multiple, often converging mechanisms, including direct or indirect involvement of hypothalamic and brainstem structures that coordinate circadian timing. Hospital-related factors such as fragmented sleep, altered sleep-wake schedules, and reduced exposure to natural light further compound this disruption. In addition, the acute stress response associated with stroke suppresses pineal melatonin synthesis, while sensory and visual pathway disturbances may impair light-mediated entrainment of the circadian system. Together, these factors contribute to circadian desynchronization and loss of normal temporal organization of neuroendocrine and autonomic functions [14,30]. In patients with acute stroke, nocturnal melatonin secretion is often markedly reduced, reflecting disruption of normal circadian signaling. This decline in melatonin has been linked to impaired sleep quality, including fragmented sleep, reduced sleep efficiency, and altered sleep architecture. Beyond its effects on sleep, diminished nocturnal melatonin is associated with autonomic imbalance, characterized by heightened sympathetic activity and reduced parasympathetic tone, which may contribute to cardiovascular instability. Clinically, lower melatonin levels in the acute phase of stroke have been correlated with poorer neurological outcomes, suggesting that circadian and neuroendocrine dysregulation may influence both recovery trajectories and overall prognosis [31-33]. Melatonin deficiency in the context of acute stroke may intensify secondary brain injury by weakening the brain’s endogenous defenses against oxidative stress and inflammation. Normally, melatonin functions as a potent antioxidant, directly scavenging free radicals and enhancing the activity of cellular antioxidant systems, while also modulating microglial activation and cytokine production to limit neuroinflammation. When melatonin levels are reduced, these protective mechanisms are compromised, allowing reactive oxygen species and pro-inflammatory mediators to accumulate. This creates a permissive environment for neuronal damage, exacerbates ischemia-induced cellular injury, and may contribute to larger infarct volumes and worse functional outcomes [28,29].

Autonomic Dysfunction and Cardiovascular Regulation

In acute ischemic stroke, injury to central autonomic networks-including the insular cortex, hypothalamus, and brainstem-disrupts the normal balance between sympathetic and parasympathetic activity. This disruption results in heightened sympathetic outflow, characterized by increased heart rate, elevated blood pressure, and enhanced catecholamine release, alongside diminished parasympathetic modulation. The shift toward sympathetic dominance not only contributes to cardiovascular instability but also influences systemic inflammatory responses, metabolic regulation, and cerebral perfusion. Such an autonomic imbalance represents a key pathway through which acute stroke can produce widespread systemic effects, linking central neural injury to peripheral physiological dysregulation [12,34]. Clinically, stroke-induced autonomic imbalance is reflected in a range of measurable cardiovascular abnormalities. Patients frequently exhibit persistent tachycardia and reduced heart rate variability, indicating diminished parasympathetic modulation and heightened sympathetic dominance. Impaired baroreflex sensitivity further compromises the body’s ability to regulate blood pressure in response to physiological changes, contributing to sudden fluctuations in systemic arterial pressure. This blood pressure lability can exacerbate cerebral ischemia, increase the risk of secondary vascular events, and challenge hemodynamic management in the acute care setting. Collectively, these cardiovascular manifestations serve as readily observable markers of autonomic dysfunction following acute stroke [34-36]. Autonomic dysfunction is especially pronounced in patients experiencing acute or severe ischemic strokes, reflecting the extent of disruption within central autonomic networks. The severity of autonomic imbalance correlates with the intensity of the neurological insult, with larger infarcts or involvement of critical regulatory regions producing more marked sympathetic predominance and parasympathetic withdrawal. This dysregulation significantly increases the risk of cardiovascular complications, including arrhythmias, blood pressure instability, myocardial injury, and sudden cardiac events. Recognition of autonomic dysfunction in the acute phase is therefore clinically important, as it not only reflects the severity of neural injury but also serves as a predictor of cardiovascular morbidity and mortality in stroke patients [22,37].

Blood Pressure Variability and Dipping Status

Blood pressure variability has emerged as a key indicator of autonomic dysfunction in acute ischemic stroke, reflecting the impaired integration of sympathetic and parasympathetic control over vascular tone. Rather than representing a static elevation or reduction in blood pressure, variability captures the dynamic fluctuations in arterial pressure over time, which can indicate loss of normal baroreflex function and autonomic regulation. Increased blood pressure variability is associated with hemodynamic instability, greater risk of secondary ischemic injury, and poorer neurological outcomes. As a clinically measurable parameter, it provides insight into the severity of autonomic imbalance and may serve as a useful biomarker for monitoring stress-related cardiovascular effects in stroke patients [38,39]. In patients with acute ischemic stroke, both short-term and circadian blood pressure variability are frequently elevated, reflecting disrupted autonomic regulation and impaired baroreflex function. Normally, blood pressure follows a circadian rhythm characterized by lower nocturnal values, known as “dipping,” but this pattern is often lost after stroke. The combination of heightened variability and loss of nocturnal dipping has been linked to adverse clinical outcomes, including poorer functional recovery, increased risk of hemorrhagic transformation, and higher mortality. These observations highlight the prognostic significance of blood pressure patterns in stroke and underscore the importance of continuous monitoring of dynamic blood pressure fluctuations as a marker of autonomic and neuroendocrine dysregulation [40-43]. Ambulatory blood pressure monitoring (ABPM) offers a continuous, dynamic assessment of blood pressure over 24 hours, capturing fluctuations and circadian patterns that are not evident during isolated office measurements. By measuring short-term variability, nocturnal dipping, and diurnal trends, ABPM provides valuable insights into autonomic regulation and cardiovascular responses following acute ischemic stroke. This approach allows clinicians and researchers to detect subtle abnormalities in blood pressure control that reflect sympathetic overactivity, parasympathetic withdrawal, and impaired baroreflex function. Consequently, ABPM serves as both a diagnostic and prognostic tool, linking vascular patterns to neuroendocrine and autonomic status and enabling more informed management strategies in the acute and subacute phases of stroke [44,45].

Interaction Among Cortisol, Melatonin, and Blood Pressure Regulation

Neuroendocrine and circadian mechanisms interact closely in regulating cardiovascular function [18,46]. Elevated cortisol levels following acute ischemic stroke can directly influence cardiovascular regulation by promoting vasoconstriction, enhancing sodium and water retention, and increasing vascular reactivity. These effects amplify systemic vascular tone and contribute to sustained elevations in blood pressure. Additionally, cortisol-mediated alterations in vascular responsiveness and endothelial function can accentuate fluctuations in arterial pressure, thereby increasing both short-term and circadian blood pressure variability. Through these mechanisms, hypercortisolemia links neuroendocrine stress activation to hemodynamic instability, highlighting its role in the pathogenesis of post-stroke hypertension and as a contributing factor to adverse cardiovascular and neurological outcomes [19,47]. In contrast to the pressor and vasoreactive effects of cortisol, melatonin has antihypertensive and sympatholytic properties that help maintain cardiovascular stability. By modulating autonomic output, melatonin reduces sympathetic tone and enhances parasympathetic activity, promoting a balanced autonomic profile. These effects contribute to the characteristic nocturnal decline in blood pressure, known as “dipping,” which supports optimal cerebral perfusion and reduces cardiovascular stress during sleep. Through its combined vascular, autonomic, and circadian actions, melatonin counteracts the hemodynamic consequences of stress-induced hypercortisolemia, highlighting its protective role in regulating blood pressure patterns and mitigating autonomic dysfunction in acute and subacute stroke [29,48].

Suppression of melatonin combined with hypercortisolemia shifts the autonomic balance toward sympathetic dominance, resulting in non-dipping or reverse-dipping blood pressure patterns frequently observed after stroke [31,42,46]. This maladaptive interaction underscores the integrated nature of neuroendocrine and cardiovascular regulation in acute cerebrovascular disease.

 Most clinical evidence linking cortisol and autonomic alterations to stroke severity and outcomes is derived from observational human studies, where associations may be influenced by confounding factors such as baseline stroke severity, systemic infections, ICU-related stressors, and medication effects. While mechanistic insights from experimental and preclinical models suggest plausible causal pathways involving HPA axis activation, neuroinflammation, and autonomic imbalance, direct causal relationships in humans remain incompletely established. Therefore, current clinical data should be interpreted primarily as associative rather than definitive evidence of causality.

Clinical implications

Prognostic Significance

Neuroendocrine markers such as cortisol, melatonin, and blood pressure variability have potential prognostic value in acute stroke [6,31,40]. Elevated cortisol levels and abnormal circadian blood pressure patterns are associated with increased stroke severity, complications, and poor functional recovery [20,41,43].

Therapeutic Considerations

Recognition of neuroendocrine dysregulation in acute ischemic stroke underscores the critical need for comprehensive management strategies that extend beyond conventional neurological care. Optimal blood pressure control is essential to minimize secondary ischemic injury and limit the impact of heightened blood pressure variability. Similarly, strategies aimed at improving sleep quality and restoring circadian rhythms can support melatonin secretion, enhance autonomic balance, and reduce sympathetic overactivity. Stress reduction interventions, whether through pharmacologic modulation of the HPA axis, behavioral measures, or environmental optimization, may further mitigate hypercortisolemia and its deleterious cardiovascular and metabolic effects. Together, these approaches emphasize a holistic, systems-based perspective in acute stroke management, targeting neuroendocrine and autonomic homeostasis to improve functional outcomes and reduce complications [44,49]. Although routine pharmacologic manipulation of stress hormones is not currently recommended in the management of acute ischemic stroke, emerging research is exploring interventions that target neuroendocrine and autonomic pathways. Chronotherapeutic strategies aim to restore normal circadian patterns, for example, through controlled light exposure or timed administration of melatonin, to support nocturnal hormone rhythms and improve sleep-wake cycles. Similarly, interventions designed to modulate autonomic function-such as beta-blockers, vagal nerve stimulation, or non-pharmacologic stress reduction techniques-are being investigated for their potential to reduce sympathetic overactivity, stabilize blood pressure variability, and protect against secondary cardiovascular complications. These approaches represent promising avenues for adjunctive therapy that could complement standard stroke care by addressing systemic neuroendocrine and autonomic dysregulation [46,50].

## Conclusions

The most available human data regarding cortisol, melatonin, autonomic dysfunction, and blood pressure variability in acute ischemic stroke are derived from observational studies. These associations may be influenced by confounding factors such as stroke severity, systemic infections, ICU-related environmental factors, medication use, and hemodynamic management strategies. Therefore, causal relationships cannot be definitively established, and findings should be interpreted within the context of these limitations.

The neuroendocrine stress response represents a central mechanism in the systemic pathophysiology of acute ischemic stroke. Acute cerebral injury triggers robust activation of the HPA axis, resulting in elevated cortisol levels, while simultaneously disrupting pineal melatonin secretion and causing marked autonomic imbalance. These interconnected processes exert wide-ranging effects on cardiovascular regulation, including blood pressure variability and loss of nocturnal dipping, modulate immune function and inflammatory responses, and influence neuronal survival and recovery. A comprehensive understanding of these mechanisms not only clarifies the complex interplay between central and peripheral responses to stroke but also emphasizes the potential utility of neuroendocrine and autonomic biomarkers, such as cortisol, melatonin, and ambulatory blood pressure patterns, in prognostication, risk stratification, and the development of individualized therapeutic strategies.
